# Detecting parallel polygenic adaptation to novel evolutionary pressure in wild populations: a case study in Atlantic cod (*Gadus morhua*)

**DOI:** 10.1098/rstb.2022.0190

**Published:** 2023-07-17

**Authors:** Brendan N. Reid, Bastiaan Star, Malin L. Pinsky

**Affiliations:** ^1^ Department of Ecology, Evolution, and Natural Resources, Rutgers University, New Brunswick, NJ 08540, USA; ^2^ Center for Ecological and Evolutionary Synthesis, Department of Biosciences, University of Oslo, PO Box 1066, Blindern, 0316 Oslo, Norway

**Keywords:** fisheries-induced evolution, convergence, parallel adaptation

## Abstract

Populations can adapt to novel selection pressures through dramatic frequency changes in a few genes of large effect or subtle shifts in many genes of small effect. The latter (polygenic adaptation) is expected to be the primary mode of evolution for many life-history traits but tends to be more difficult to detect than changes in genes of large effect. Atlantic cod (*Gadus morhua*) were subjected to intense fishing pressure over the twentieth century, leading to abundance crashes and a phenotypic shift toward earlier maturation across many populations. Here, we use spatially replicated temporal genomic data to test for a shared polygenic adaptive response to fishing using methods previously applied to evolve-and-resequence experiments. Cod populations on either side of the Atlantic show covariance in allele frequency change across the genome that are characteristic of recent polygenic adaptation. Using simulations, we demonstrate that the degree of covariance in allele frequency change observed in cod is unlikely to be explained by neutral processes or background selection. As human pressures on wild populations continue to increase, understanding and attributing modes of adaptation using methods similar to those demonstrated here will be important in identifying the capacity for adaptive responses and evolutionary rescue.

This article is part of the theme issue ‘Detecting and attributing the causes of biodiversity change: needs, gaps and solutions’.

## 1. Introduction

Biodiversity is changing rapidly in response to human activity [1]. When faced with accelerating environmental change in the Anthropocene, many wild populations may only be able to persist through evolutionary adaptation to novel conditions [2,3]. Such evolutionary responses to recent change have been suggested in multiple taxa, including birds [4,5], fish [6], mammals [7], insects [8] and plants [9]. Proving that responses have been evolutionary rather than the result of phenotypic plasticity, however, has often been difficult in the wild [10].

The capacity for contemporary evolution depends on the amount of existing genomic variation and on the genomic architecture of the trait under selection [11]. Highly polygenic traits may have a greater capacity for evolutionary response to novel conditions [12,13]. Since a large number of loci may underpin these traits, however, genetic redundancy (or the degree to which multiple combinations of different alleles can produce the same phenotype; [14]) may be high, such that different loci can contribute to a similar phenotypic response across populations [15]. If the same loci contribute to the evolution of a similar trait value in different populations, the evolutionary genetic response is considered parallel. The degree to which polygenic evolutionary responses are parallel or non-parallel will depend on a number of factors, including the frequency of alleles contributing to the selective response in the founding populations, the degree to which their phenotypic effects are redundant, and the distance to a novel trait optimum [14]. Empirical studies of recent repeated adaptation have shown evidence of both parallel genetic responses [16] and non-parallel responses [17,18].

The genetic signatures of evolutionary adaptation, and the methods used to detect these signatures, depend on the genetic architecture of the trait and the data available. For traits controlled by a few loci, selection will result in distinct genomic sweeps characterized by large changes in frequency of the loci responsible for adaptation as well as nearby loci [12,19]. When spatial or temporal genome-scale genetic data are available, regions influenced by sweeps can be identified as outliers with atypically high genetic differentiation [20]. For more polygenic architectures, allele frequency changes will be more subtle and will be spread across a large number of loci, rendering tests for outliers less useful [21]. If trait data are available, genome-wide association studies (GWAS) may be able to identify loci under selection, although trait data are not always available and GWAS may be of limited utility when the trait architecture is highly polygenic [22]. Recently, a framework for detecting highly polygenic responses to selection from covariance in genome-wide allele frequency change across temporal or spatial replicates has been developed [23] and applied to evolve-and-resequence studies [24]. However, this method has not yet been applied in wild populations.

Studies of contemporary adaptation to novel environments in the wild have found that evolutionary responses can be mediated by a wide range of genomic architectures, ranging from single loci of large phenotypic effect to whole-genome polygenic architectures with many loci of very small effect [17]. When survival in altered environments is strongly controlled by a single locus, adaptive responses may depend on the presence of a particular allele [25,26]. Recent adaptation to freshwater environments in threespine sticklebacks has been mediated by a small number (less than 20) of genomic regions recycled across multiple instances of adaptation, producing parallel genetic responses in freshwater populations [27,28]. Evolution of life-history traits (including timing of reproduction and maturation) may be particularly important in determining the response to climate change and other novel selection pressures [29,30]. Since life history is often considered to be a composite character bound to multiple fitness-related traits, life-history evolution is often assumed to be highly polygenic, with many loci of small effect rather than a few loci of large effect contributing to quantitative changes in traits [31,32], although some life-history traits are controlled by only one or a few loci (e.g. migration timing in Pacific salmonids; [26,33,34]). Given the difficulties inherent in correctly detecting and attributing highly polygenic adaptive responses, studying contemporary evolution in polygenic life-history traits may require novel methods.

Here, we use the approach developed by Buffalo & Coop [24] to investigate the evidence for parallel polygenic adaptation to fishing in Atlantic cod (*Gadus morhua*). Cod were subject to intense fishing pressure in the mid-twentieth century, resulting in both a steep population decline and a phenotypic shift in life history toward smaller size at maturity and lower age at reproduction [35,36]. These responses were observed in parallel across both Northeast Atlantic and Northwest Atlantic stocks [37]. A recent study using temporal genomic data from Northeast and Northwest Atlantic populations before and after exploitation [38] found that despite population declines, Atlantic cod have retained much of their pre-decline genetic variability. Additionally, there was scant evidence for dramatic sweeps in allele frequency characteristic of adaptation via a few genes of small effect. One possibility is that phenotypic plasticity explains the developmental changes, perhaps through socially mediated developmental processes that are common in fishes [35,39–41]. Polygenic selection, however, also remains a possible explanation for the similar response to overfishing observed in these populations. Differentiating between these two possibilities has been difficult. However, spatial and temporal replication can be particularly important for determining whether evolution occurs via polygenic responses [14]. Ultimately, evolution is a change in allele frequencies through time, and some of the clearest evidence for evolutionary change in other systems has come from temporal genomic approaches [42–44].

We re-visit the genomic data from Pinsky *et al*. [38], which includes data from two cod populations in the Northeast and Northwest Atlantic sampled over a span of 100 years, using the covariance method developed by Buffalo & Coop [24] to test whether Atlantic cod exhibit a signature of parallel polygenic selection. We hypothesized that parallel polygenic selection would generate positive covariance in allele frequency change across the two sampled populations. We examine whether covariance differs across genomic regions (chromosome-level linkage groups and chromosomal inversions), and we use simulations to evaluate whether neutral processes (demographic change or gene flow) or background selection could generate comparable signals of covariance in allele frequency change. This work demonstrates the utility of novel methods for detecting recent parallel adaptation and deepening our understanding of how wild populations and species can respond to novel selective pressures.

## 2. Methods

### (a) Cod single nucleotide polymorphism data and data filtering

**Figure 1.**
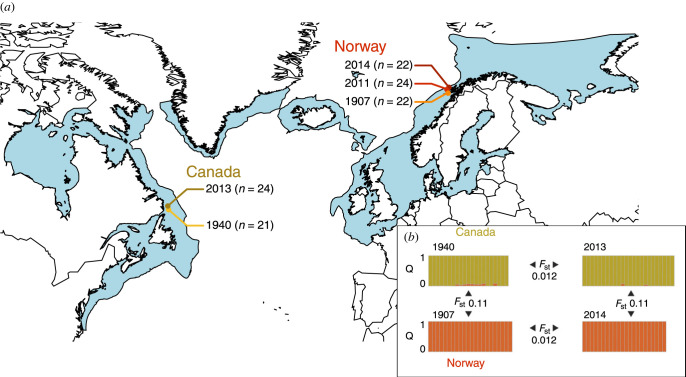
Map showing sampling scheme and divergence among populations (adapted from Pinsky *et al.* [38]). (*a*) Sampling locations and times. Distribution of Atlantic cod (dark blue) is shown based on UN FAO data (https://www.fao.org/fishery/geonetwork/srv/eng/catalog.search#/metadata/fao-species-map-cod). (*b*) Population assignment for each individual (with proportion of inferred ancestry *Q* shown as coloured bars) for Canada (1940 and 2013 samples) and Norway (1907 and 2014 samples) along with overall temporal and spatial *F*_ST_ values between these samples.

We used a SNP dataset generated by Pinsky *et al*. [38] from 113 Atlantic cod samples. The dataset includes individuals from both the Northwest Atlantic (Canada) and the Northeast Atlantic (Norway) sampled at five discrete time points (figure 1*a*). To summarize the bioinformatic methods briefly, shotgun sequence data were aligned to a reference genome from a Northeast Atlantic cod (version gadMor2) and stringently filtered to remove potentially erroneous variants that could be caused by mapping errors or DNA damage in historic samples. The final dataset consisted of 346 290 called SNPs [38].

**Figure 2.**
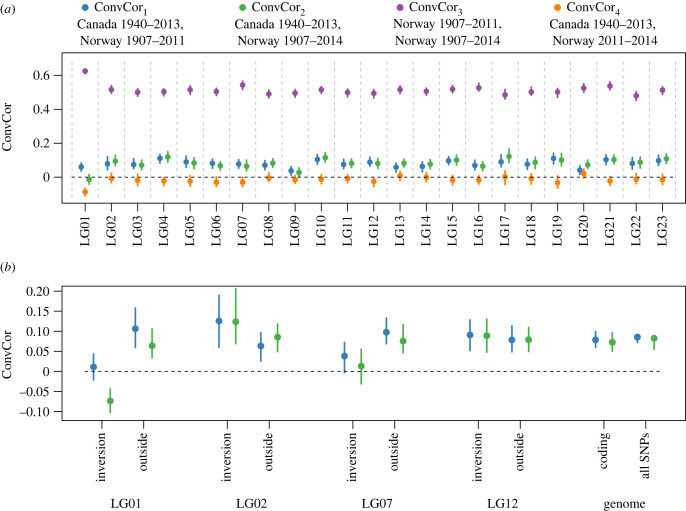
Empirical convergent correlation values from the filtered dataset. ConvCor_1_ (Canada 1940–2013, Norway 1907–2011) is shown in blue, ConvCor_2_ (Canada 1940–2013, Norway 1907–2014) in green, ConvCor_3_ (Norway 1907–2011, Norway 1907–2014) in purple, and ConvCor_4_ (Canada 1940–2013, Norway 2011–2014) in orange. Points represent the overall value, and lines represent bootstrap 95% confidence interval. (*a*) ConvCor values by linkage group. (*b*) Convergent correlations for groups of loci inside and outside known genomic inversions, as well as for SNPs in coding regions and all SNPs overall.

Although this SNP dataset (referred to hereafter as the ‘original’ dataset) was stringently filtered, some differences between the historical and modern data remained, including lower genotype quality and higher levels of missing data in historic samples for putative outlier SNPs compared to the rest of the dataset [38]. To address these potential differences, we created a second dataset further filtered for quality and missing data (hereafter the ‘filtered’ dataset). We first used vcftools v. 0.1.17 [45] to remove genotypes with Phred-scaled quality scores less than 30. We then assessed the level of missing data across each sample. As the proportion missing was highest for individuals in the 1940 Canada sample, we identified loci with less than 40% missing data across individuals in this sample and kept only these loci across all individuals.

### (b) Assessing evidence for parallel adaptation

We calculated sample-level allele frequencies at each locus in the original and filtered datasets for each of the five temporal samples from the Northwest and the Northeast Atlantic using plink v. 2.0 [46]. We then calculated the change in allele frequency between 1940 to 2013 for the Northwest Atlantic and between 1907 and either 2011 or 2014 for the Northeast Atlantic. To assess evidence for parallel adaptation, we adapted the ‘convergent correlation’ statistic described by Buffalo & Coop [24]. Covariance in allele frequency change is taken as evidence of shared response to selection (both direct selection of loci that affect the trait under selection and linked selection of loci that are physically near the loci under selection). This statistic was originally applied to allele frequency changes over the same time interval in replicated experimental populations subjected to a novel selection pressure. As identical temporal sampling intervals were not available for the cod dataset, we used the irregular time points available. We calculated the correlation as:

$$\mathrm{ConvCor}(\Delta s1,\Delta s2)=\mathrm{cov}(\Delta s1,\Delta s2)/\sqrt{\left(\mathrm{var}(\Delta s1)*\mathrm{var}(\Delta s2)\right)},$$

where Δs1 and Δs2 represent vectors of allele frequency change between two time points for a given population. We conducted all calculations using R v. 4.2.0 [47].

We calculated this statistic for a number of comparisons. To measure covariance in allele frequency change across the Northwest and Northeast Atlantic populations, we calculated ConvCor_1_ (Canada 1940–2013, Norway 1907–2011) and ConvCor_2_ (Canada 1940–2013, Norway 1907–2014). If parallel evolution occurred, we expected these to show positive correlation. We also calculated two other statistics as controls. As a positive control, we calculated ConvCor_3_ (Norway 1907–2011, Norway 1907–2014), which is the covariance between measured temporal allele frequency change for the two contemporary Norway samples. Since the contemporary samples were taken roughly within the same generation, covariance should be high and we expected this statistic to be large and positive as long as allele frequency measurements are relatively accurate and unbiased. As a negative control, we calculated ConvCor_4_ (Canada 1940–2013, Norway 2011–2014). Measured allele frequency change between the two contemporary Norway samples should mainly correspond to sampling variation, and since there should be little covariance between this and temporal allele frequency change in Canada, this statistic should be close to zero.

The cod genome contains a number of inversions with suppressed recombination among inverted haplotypes [48,49]. These regions act as ‘supergenes’ and have been implicated in differences among cod migratory ecotypes [50]. As these inversions may be under different selection pressures than the rest of the genome and are expected to act in a manner similar to one long locus rather than as independent loci, we also evaluated the ConvCor_1_ and ConvCor_2_ statistics within specific known inversions, within all known inversions, and outside of known inversions. We used the ‘high LD regions’ identified in Matschiner *et al*. [50] to define inversions on linkage groups 1, 2, 7 and 12. We also calculated each ConvCor statistic for each linkage group separately, as well as for all SNPs located within coding regions based on the annotated gadmor2 genome. We estimated 95% bootstrap confidence intervals by resampling the loci used to calculate each ConvCor statistic 100 times with replacement and re-calculating the statistic.

### (c) Simulations

Buffalo and Coop's convergence correlation statistic was developed for replicated evolve-and-resequence experiments. Natural populations differ notably from these in several ways, including the potential for migration among populations. To examine the potential for migration to create a false signal of allele frequency covariance among populations without parallel adaptation, we conducted simulations of allele frequency change over time in two populations experiencing gene flow. Forward simulations were conducted in SLiM v. 4.0 [51] using non-Wright-Fisher mode. We used parameters mimicking the known history of cod populations in the Atlantic. The simulations began with a single population representing the common ancestor of modern Atlantic cod populations with a population size of *N*_e_ = 7000 that corresponded to the Pleistocene minimum population size estimated by Matschiner *et al*. [50]. This population was simulated for 57 400 generations (roughly 574 000 years assuming 10 years per generation) and then split into two subpopulations (subpop1 and subpop2), corresponding to the split between the Northwest and the Northeast Atlantic cod populations [50]. The two subpopulations then grew at a constant rate over 6530 generations to a final size of *N*_e_ = 35 000 corresponding to recent population size of the Northeast Atlantic populations estimated by Pinsky *et al*. [38]. After the split, migration between the two subpopulations was allowed to occur, with a proportion of individuals in each population migrating to the other population each generation. The proportion of individuals migrating per generation (the migration rate) was varied over five orders of magnitude, from 10^−2^ to 10^−3^, 10^−4^, 10^−5^ and 10^−6^.

We simulated a single 5 Mb genomic region, roughly corresponding to the ‘callable bases’ for a single cod chromosome in Pinsky *et al*. [38]. Due to the reduced size of the simulated chromosome relative to the size of an actual cod chromosome (roughly six times smaller), to preserve a realistic probability of recombination among genomic regions within the simulated chromosome we set the recombination rate to 1.5 × 10^−7^, approximately six times higher than the generally assumed vertebrate recombination rate of 1 × 10^−8^ per base. We used a mutation rate of 1.64 × 10^−8^ per base per generation previously estimated for Atlantic cod [50]. Two ‘historic’ samples (VCF files) of 20 individuals from subpopulation 1 and subpopulation 2 were taken at 6525 and 6530 generations after the split (corresponding to the historic samples taken from Norway and Canada, respectively). Finally, three ‘contemporary’ samples were also taken. Two additional samples of 20 individuals each were taken from subpopulation 1 and one additional sample of 20 individuals was taken from subpopulation 2 at 6540 generations after the split, corresponding to the contemporary samples taken from Norway and Canada, respectively. Twenty replicate simulations were conducted for each migration scenario. To match filters applied to the empirical dataset, we filtered the simulated datasets to remove any loci with minor allele frequency less than 0.05 and any loci with more than two alleles. We calculated Weir and Cockerham's *F*_ST_ using vcftools between the two populations using both the historic samples and the contemporary samples, as well as between the two time points for each population. We also calculated the four ConvCor statistics described above using the corresponding simulated samples. We compared simulated *F*_ST_ values to empirical *F*_ST_s calculated by Pinsky *et al*. [38] (figure 1*b*) and we compared simulated ConvCor statistics to the empirical statistics calculated here.

Parallel polygenic selection is most likely to occur if populations share adaptive variants that are present at high frequencies [14]. Covariance in allele frequency change could also be produced by background selection on shared deleterious variation [24], meaning that if strongly deleterious variants arose prior to the split between the two populations and persisted to the present, these variants could also produce a similar signal of covariance in allele frequency change. To evaluate the potential distribution of allele frequencies and ages for different types of variants, we conducted an additional simulation that included neutral mutations, deleterious mutations, and mutations under stabilizing selection. We parameterized this simulation with the same set of demographic parameters used for the neutral simulation, and we used a migration probability of 10^−4^. We simulated deleterious mutations as completely recessive, with fitness effects following a gamma distribution with a mean of −0.05 and a shape parameter of 0.5 (after [52]). For mutations under stabilizing selection, we modified the quantitative trait loci (QTL) parameterization from SLiM's template for simulating polygenic selection, in which mutations have an average phenotypic effect of 0 and a variance of 1, and selection maintains a phenotype near an optimum value of 0. The same total mutation rate (1.64 × 10^−8^) was used, with each type of mutation equally likely to occur. To capture the full spectrum of all mutations, we did not apply a minor allele frequency filter to this simulated dataset. We identified how many mutations segregating in the present time of each type (under neutral, deleterious or balancing selection) occurred before and after the split between the two populations as well as the mean frequencies of mutations in the present of each type occurring before and after the split.

## 3. Results

### (a) Dataset and filtering

The original dataset consisted of 346 290 SNPs, with historical samples tending to exhibit higher levels of missing data than contemporary samples (electronic supplementary material, figure S1a). The filtered dataset contained 112 082 loci with roughly equal proportions of missing data across samples (electronic supplementary material, figure S1b).

### (b) Assessing evidence for parallel adaptation

The genome-wide convergence correlations across the Atlantic were positive and similar across the two contemporary Norway timepoints for the unfiltered dataset (ConvCor_1_= 0.139, ConvCor_2_ = 0.119) and the filtered dataset (ConvCor_1_ = 0.085, 95% bootstrap CI = 0.08 to 0.092; ConvCor_2_ = 0.082, 95% bootstrap CI = 0.077 to 0.088). The ConvCor_1_ and ConvCor_2_ values for each linkage group were uniformly positive for the unfiltered dataset (range: 0.0871 to 0.317) and there was only one negative value in the filtered dataset (range: −0.013 to 0.123; figure 2*a*; electronic supplementary material, figure S2). The negative value was for linkage group 1, which has a large inversion. Particularly low and high values for the filtered and unfiltered datasets, respectively, were associated with the inversion in linkage group 1. On both linkage groups 1 and 7, the ConvCor_1_ and ConvCor_2_ statistics were quite low inside the inversion, but higher outside for the filtered data (figure 2*b*; electronic supplementary material, figure S3). ConvCor statistics calculated for coding SNPs did not differ substantially from the genome-wide statistics for the filtered dataset but were lower than the genome-wide statistics for the original dataset (figure 2*b*; electronic supplementary material, figure S3).

As expected for the positive control, ConvCor_3_ was high for both the filtered dataset (0.519, 95% bootstrap CI = 0.514 to 0.523) and for the unfiltered dataset (0.712, 95% bootstrap CI 0.71 to 0.715) (figure 2*a*; electronic supplementary material, figure S2). As expected for the negative control, ConvCor_4_ was close to zero, exhibiting slightly negative values for the filtered dataset (−0.026, 95% bootstrap CI −0.019 to −0.031) and the unfiltered dataset (genome-wide = −0.042, 95% bootstrap CI −0.019 to −0.031) (figure 2*a*; electronic supplementary material, figure S2).

### (c) Simulations

Spatial *F*_ST_ values between subpopulations for both the historical and contemporary samples generated by simulations varied from near zero (for migration rates of 10^−2^ and 10^−3^) to 0.08–0.12 (for migration rates of 10^−5^ and 10^−6^). The *F*_ST_ from these lower migration rates approximately matched the rates observed in empirical cod populations (*F*_ST_ = 0.11; electronic supplementary material, figure S4). Simulated temporal *F*_ST_ values between time points within a subpopulation, on the other hand, were close to zero for all migration scenarios, suggesting that very little genetic drift is expected for these populations given their size and the number of generations elapsed between sampling points. Observed temporal *F*_ST_ values were approximately 0.012 for both populations, which was larger than any of the simulated values (electronic supplementary material, figure S4).

**Figure 3.**
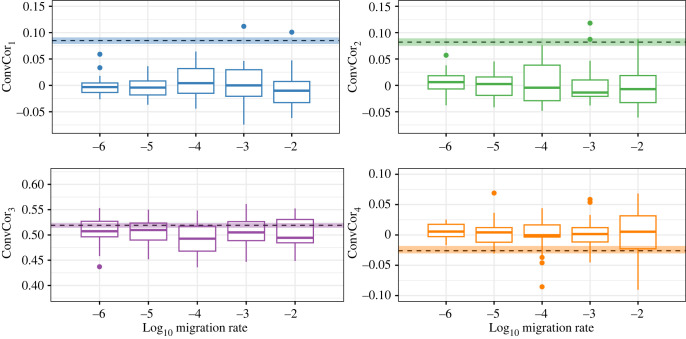
Box-and-whisker plots showing distribution of simulated values for the four convergent correlation statistics. Migration rate is the proportion of simulated individuals migrating between populations in each generation. Dotted lines show genome-wide values and coloured bands show the 95% bootstrap confidence interval calculated from the filtered empirical dataset.

The ConvCor_1_ and ConvCor_2_ statistics calculated for simulated data were on average near zero but demonstrated substantial variability, especially when migration rates were higher (≥10^−3^). The observed genome-wide ConvCor_1_ and ConvCor_2_ statistics, however, were larger than any of the simulated statistics for both the unfiltered and filtered datasets at the lower migration rates (≤10^−5^) that were consistent with observed *F*_ST_ (figure 3). Simulated values for ConvCor_3_ were approximately 0.5 across migration scenarios, similar to values observed for the filtered cod dataset but lower than values for the unfiltered dataset (figure 3). Simulated values for ConvCor_4_ were close to zero, and observed values were similar or slightly lower (figure 3).

The simulation including neutral, deleterious and QTL variants indicated that most segregating variants were recent. Of the mutations (all three variant types) still segregating at the end of the simulation, 2.5% had originated before the split between the two populations. The fraction of segregating deleterious recessive mutations originating before the split was lower (only 1.1%). For the other types of mutations, 4.8% of segregating neutral mutations had originated before the split, and 1.2% of QTL variants had originated before the split (electronic supplementary material, figure S5a). Mutations originating before the split tended to exhibit a much higher allele frequency at the end of the simulation than mutations originating after the split for each type of variant. The mean frequency of neutral mutations originating before the split was 0.247, while the mean frequency of mutation originating after the split was only 2.38 × 10^−3^ (electronic supplementary material, figure S5a). The equivalent frequencies for QTL variants was 0.208 (before-split) and 8.88 × 10^−4^ (after-split; electronic supplementary material, figure S5a), and for deleterious variants equivalent frequencies were 0.083 (before-split) and 0.001 (after-split). While simulated mutations originating from before the split comprised a small proportion of all segregating mutations, they made up the majority (86.3%) of segregating variants with allele frequencies greater than 0.05 at the end of the simulation. Segregating variants in the empirical dataset (which was filtered to exclude alleles with minor allele frequencies less than 0.05) exhibited a similar pattern, with a majority (76.3%) of SNPs segregating in all populations. The distribution of fitness effects of deleterious mutations originating before the split was highly skewed toward zero compared to the distribution of effects originating after the split, indicating that shared deleterious mutations had weaker effects on fitness compared to more recent unshared mutations (electronic supplementary material, figure S5b). QTL mutations originating before the split also tended to be of smaller phenotypic effect sizes than mutations originating after the split (electronic supplementary material, figure S5c).

## 4. Discussion

Despite rapid phenotypic change in Atlantic cod associated with intensive fishing, clear genomic evidence for evolutionary adaptation has been elusive to date [53]. Here, we found evidence for parallel evolutionary responses to novel selection pressures across two cod populations. Cod populations in the Northeast and Northwest Atlantic showed remarkably consistent positive covariance in allele frequency change over the last seven to ten decades regardless of genomic linkage group, suggesting that an evolutionary response to fishing was mediated by allele frequency changes across many loci of small effect. This finding is consistent with the trait under selection being a highly polygenic quantitative trait, in line with the architecture of many other life-history traits [32].

The accumulated support for fisheries-induced evolution, and evolution in harvested populations in general, has thus far mainly consisted of abundant evidence for selection on and phenotypic change in life-history traits, with comparatively little molecular evidence for changes in specific genes [37]. Many populations showing evidence for fisheries-induced evolution, including cod, also show potential for reversibility of phenotypic change when the pressure of selective harvesting is removed [35,38,54]. A highly polygenic architecture for fisheries-induced evolution, as suggested in this study, provides a potential basis for reconciling these observations. As is the case for many traits implicated in local adaptation in fish, evolution of traits under fisheries-induced evolutionary pressure may be mediated by small changes in many alleles with high levels of standing genetic variation [55], meaning that these evolutionary responses can occur rapidly and have the potential for reversal when fishing pressure is removed.

While our results are consistent with a highly polygenic response to fisheries-induced selection, it is important to note that definitive causal attribution is difficult with the current data. Cod populations are responding to a number of changes in the marine environment, including changes in climate, biotic interactions and other factors [56,57]. Covariance in allele frequency change may represent a shared genetic response to one of these other factors or to multiple factors (including fishing) combined. A more definitive attribution of the causes is usually explored with experiments, but these are difficult in long-lived species like cod. Attributing polygenic responses is also more difficult than attributing responses mediated by one or a few traits because oligogenic responses can often be traced back to particular genes with functions related to the observed evolutionary response [25,43]. Alternatively, sampling multiple populations subject to a gradient of fishing pressure and environmental change over multiple time points could enable more robust tests of fisheries-induced evolution in a causal modelling framework [58].

Life-history traits such as age at maturity can also exhibit genomic architectures that are not genome-wide but rather highly localized. Clusters of genes, or ‘supergenes’, residing within genomic inversions have been shown to underlie divergence between stationary and migratory ecotypes in cod [48,49] as well as other ecologically important traits in Atlantic silversides [59], sunflowers [60] and butterflies [61]. While theory predicts that supergene complexes within inversions may be particularly important in parallel evolution [62], the trans-Atlantic response to fishing does not in this case appear to be particularly strongly associated with inversions, and correlations in allele frequency change within inversions tended to be somewhat weaker than the genome-wide trend in the filtered dataset. Strong directional selection can produce both low levels of within-population diversity and high levels of differentiation among populations within inversions (as in silversides; [63]), and this may reduce the role of these inversions in parallel adaptation in cod. Investigating the role of inversions in other instances of fisheries-induced evolution would, however, still be a fruitful avenue for future research.

Covariance in allele frequency change over time could conceivably be produced by the joint action of migration and drift as well. Without migration, drift will tend to produce divergent allele frequency changes across populations, but with sufficient migration, allele frequencies in separate populations will tend to change in the same direction. The neutral genetic simulations performed here suggest, however, that the observed genetic differentiation among populations is consistent with very low migration rates (less than 10^−5^ probability of an individual migrating between populations per generation). This inferred rate of migration is consistent with population assignment and clustering analyses conducted for these populations, which suggested strong differentiation and little evidence of admixture among the Northeast and Northwest Atlantic populations [38,50,57]. Neutral simulations suggested that false positives for the convergence correlation statistic are unlikely to be generated by migration at the low rates experienced by Northeast and Northwest Atlantic cod. Although observed values for spatial divergence were consistent with expectations based on cod demographic history, observed values for temporal divergence were higher than expected under all migration scenarios. The observations of high temporal divergence could potentially be explained by genome-wide allele frequency change due to a novel selection pressure, which would be consistent with our finding of covariance in allele frequency change. In general, neutral simulations are valuable for generating expectations for change over time without selection. Incorporating selective pressures into the simulation framework used here can also help produce expectations for non-neutral scenarios. For example, incorporating deleterious mutations into our simulation framework indicated that strongly deleterious mutations would likely not be shared between populations, suggesting that background selection on shared deleterious variants is unlikely to explain the observed results. However, since conducting more realistic non-neutral simulations would require considerably more knowledge of the genomic architecture of traits under selection and past and present history of selection on those traits, we consider these simulations to be a first step toward fully understanding the evolutionary trajectories of cod genomes. Our simulations also used a relatively simple model of recombination with only a single simulated chromosome to increase computational efficiency. More realistic simulations incorporating variability of recombination rate across the genome would be useful for better understanding the effects of linked selection and explaining variation in the observed covariance in allele frequency change across the genome and within chromosomal inversions.

It is also important to note that, although the original data were stringently filtered and subjected to additional filtres in this study, artefacts in historical data could have influenced our results. Errors in historic data due to DNA degradation can be random (such as introducing singleton alleles) or systematically biased, such as increased rates of transversions or reference bias [64,65]. While random changes are highly unlikely to produce covariance among change across populations, systematic biases could. The original dataset was filtered to remove transversions and minimize reference bias as a factor [38], meaning that these sources of systematic bias were minimized, but careful attention to systematic bias should always be examined if covariance in allele frequency change is of interest.

The multi-population temporal method used here holds promise for detecting polygenic evolutionary change in the wild. Detecting such responses in the past has been very difficult. We note, however, that this approach is less likely to work in cases involving extremely polygenic traits exhibiting high genetic redundancy. Such redundancy can produce non-parallel responses across populations because the same phenotype change can be produced by independent locus sets [21]. When responses are highly non-parallel, the convergent correlation method would likely not detect a signal of convergent allele frequency change. The likelihood of non-parallel responses will depend on multiple factors, including the number of loci responsible for a trait (more loci generally meaning a higher degree of redundancy and a higher probability of non-parallel responses) as well as the frequency of adaptive alleles in the population, with shared high-frequency alleles increasing the likelihood of repeatable evolutionary responses. By contrast to experimentally generated populations in evolve-and-resequence experiments originally used to develop Buffalo and Coop's covariance statistics, real populations also have complex, long-term histories of divergence and demographic change that could affect the potential for redundant or non-parallel responses [66]. Populations that have diverged in the more distant past will be more likely to lose shared quantitative loci by drift and gain population-specific loci through mutation, meaning that the proportion of shared QTL will likely decrease with split times further in the past [67]. The simulation of QTL conducted here suggested that many of these loci may indeed be recent mutations. However, these simulations also suggested that recent mutations will likely be present at very low frequencies and that shared QTL that originated before the populations split will be present at much higher frequencies and more available to selection by novel environments. These simulations therefore suggest that repeated polygenic adaptation via high-frequency alleles could explain the signal of covariance in allele frequency change observed in trans-Atlantic cod populations despite their past divergence. Over longer time periods, however, covariance in allele frequency change across populations may decay [24,68] as alternate loci contribute to long-term adaptation in different populations. We anticipate that this could also occur in cod, particularly as more recent unshared mutations present at low frequencies begin to exhibit larger changes in allele frequencies. Even when the same loci are under selection across populations, divergence may obscure the signal of covariance in allele frequency change across the genome since recombination will break up associations shared among populations between causative loci and linked neutral loci over time [69]. While the particular sampling scenario examined here (parallel selection in two divergent populations) may not be possible for many systems, if more than two time points are available for a single population similar statistics can also be calculated [24]. Redundancy and loss of linkage will not be as much of a problem for multi-temporal sampling schemes as long as the same causative loci continue to contribute to phenotypic change through time. Overall, assessing covariance in genome-wide allele frequency change is a promising means of detecting polygenic responses to novel selection pressures in the wild, and using these methods to assess past selective responses and the possibility for future responses will be an important component of conservation management in an evolutionary framework.

## Data Availability

Scripts used to conduct the analyses can be found at https://github.com/pinskylab/codPolyEvol and are archived as a Git repository through Zenodo at https://doi.org/10.5281/zenodo.7612393 [70]. The original VCF files used to calculate allele frequencies can be downloaded at https://doi.org/10.6084/m9.figshare.22006988. The data are provided in electronic supplementary material [71].
